# Supplementary material for: The genome sequence of the Coxcomb Prominent, Ptilodon capucinus (Linnaeus, 1758)
Source: Wellcome Open Res. 2023 Jun 14;8:242. [Version 1] doi: 10.12688/wellcomeopenres.19539.1 (PMC11109679; doi:10.12688/wellcomeopenres.19539.1)
Supplement: Peer review history

# Reviewer report: Reviewer response for version 1

**DOI:** 10.21956/wellcomeopenres.21643.r74963

**Published:** 2024-05-21

**Authors:** James Mallet

**Affiliations:** 1 Harvard University, Cambridge, Massachusetts, USA

There's nothing much to say. The paper reports a genome assembly of the relevant species. Most of the reads mapped to "31 chromosomal pseudomolecules," as they should, given that the mode of chromosome numbers in the Lepidoptera is 31. The mitochondrial genome was also assembled successfully. As far as I can see the identity of the chromosomes was not verified against those of other species. The Z chromosome was reportedly identified as OU611807.1, but it is not explained how, since the sequence data was from a male specimen, and the Z would therefore normally be at the same coverage as autosomes. However, this seems typical for the work in this series of papers, and the chromosomes would certainly be identifiable via comparative study in the future. 

 Overall, an impressive and successful assembly which will be useful for further and ongoing work on the comparative genomics of Lepidoptera.

Are sufficient details of methods and materials provided to allow replication by others?

Yes

Is the rationale for creating the dataset(s) clearly described?

Partly

Are the datasets clearly presented in a useable and accessible format?

Yes

Are the protocols appropriate and is the work technically sound?

Yes

Reviewer Expertise:

Comparative genomics, evolutionary biology of the Lepidoptera

I confirm that I have read this submission and believe that I have an appropriate level of expertise to confirm that it is of an acceptable scientific standard.

# Reviewer report: Reviewer response for version 1

**DOI:** 10.21956/wellcomeopenres.21643.r74958

**Published:** 2024-03-14

**Authors:** Vlad Dincă

**Affiliations:** 1 University of Oulu, Oulu, Finland

In this study, the authors have successfully generated a chromosome-level genome assembly of a male *Ptilodon capucinus* (Lepidoptera, Notodontidae). The mitochondrial genome has been assembled as well.

 Although I am not an expert in several of the methodological approaches used, as far as I can tell, the manuscript is technically sound and uses methodologies and pipelines that are fairly well-established through use by the Sanger Institute.

 The genome appears to be of high quality and has a BUSCO v5.3.2 completeness of 98.7%.

 It is a pity that the W sex chromosome is lacking (a male was sequenced).

 Reference genomes such as this one represent a valuable resource for the scientific community and each new addition provides new opportunities for research.

 A few additional comments are included below.

 Numerous sources list the species as “ *Ptilodon capucina*”. Although this study is obviously not focused on nomenclatural aspects, I wonder whether a short explanation (in the Background section) of this name discrepancy is necessary (perhaps it is a case of gender agreement issue, which is the subject of notable debate in some groups – e.g. European butterflies). Briefly addressing this may be useful given that we are dealing with the first reference genome for this species and it is good to avoid any potential ambiguities, even if slight.

 I see Ptilodontinae is listed as subfamily. However, various sources seem to list this species under Notodontinae. I tried to find more information about this, but I was not able to find much, at least not based on DNA data. What I could find does not seem to provide strong support for Ptilodontinae, although these studies used either limited DNA data (Kobayashi & Nonaka 2016), or were not focused on this issue (St Laurent R, et al. 2023 [Ref 1]).

 It is possible that I may have missed some key study (Notodontidae are not my main area of taxonomic expertise). Nevertheless, I wanted to mention this subfamily aspect that may also need a short clarification/mention in the Background section.

 I wonder how much is known in terms of genetic structure for *P. capucinus*. Even if limited data is available (e.g. mtDNA, a few nDNA markers etc), if any notable aspect is known (e.g. diverged lineages), it could be worth briefly mentioning in the Background.

 Finally, it may also be good to mention whether *Wolbachia* has been detected in the sample analysed (I assume not, but it may be useful to mention that).

https://www.academia.edu/32479616/Molecular_phylogeny_of_the_Notodontidae_Subfamilies_inferred_from_28S_rRNA_sequences_Lepidoptera_Noctuoidea_Notodontidae

**References**

 Kobayashi, H., Nonaka, M. (2016) Molecular phylogeny of the Notodontidae: subfamilies inferred from 28S rRNA sequences (Lepidoptera, Noctuoidea, Notodontidae). *Tinea*, 23, 1–83.

Are sufficient details of methods and materials provided to allow replication by others?

Yes

Is the rationale for creating the dataset(s) clearly described?

Yes

Are the datasets clearly presented in a useable and accessible format?

Yes

Are the protocols appropriate and is the work technically sound?

Yes

Reviewer Expertise:

Lepidoptera speciation, taxonomy and phylogeography, with a focus on Western Palearctic butterflies.

I confirm that I have read this submission and believe that I have an appropriate level of expertise to confirm that it is of an acceptable scientific standard.

## References

[1] : Phylogenetic systematics, diversification, and biogeography of Cerurinae (Lepidoptera: Notodontidae) and a description of a new genus. *Insect Systematics and Diversity* .2023;7(2) : 10.1093/isd/ixad004 10.1093/isd/ixad004
